# Impact of COVID-19 hospitalization on December 2022 hospital staff in Sichuan, China

**DOI:** 10.1097/MD.0000000000043784

**Published:** 2025-08-08

**Authors:** Jing Pu, Mi Fan, Binwei Lin, Xiaobo Du

**Affiliations:** a Department of Nursing, Mianyang Central Hospital, Mianyang, China; b Department of Oncology, Affiliated Hospital of North Sichuan Medical College, Nan Chong, China; c Department of Oncology, Mianyang Central Hospital, Mianyang, China.

**Keywords:** COVID-19, diagnosis, hospital staff, symptoms, treatment

## Abstract

It is unclear how hospital staff infected with coronavirus disease (COVID-19) are diagnosed and treated, and the impact of COVID-19 on hospitals. This study investigated the symptoms, diagnosis, treatment, and impact of the COVID-19 pandemic on hospital workers in December 2022. Overall, 1464 hospital workers were surveyed by an anonymous investigator using the Questionnaire Star APP, File S1, Supplemental Digital Content, https://links.lww.com/MD/P619 (15 items). Among participants, 87.64% were diagnosed with COVID-19 and 78.79% had been infected within 15 days. Higher non-infection rate occurred in males (16.15%), aged >50 years (31.77%), and service workers (32.02%). Of the 1283 infected patients, 14.50%, 40.45%, and 45.05% were diagnosed by nucleic acid detection, antigen testing, and self-diagnosis based on symptoms, respectively. Predominant symptoms included cough (88.54%), fever (79.42%), body pains (73.03%), and expectoration (70.30%). Individuals experiencing symptoms lasting <5 days, 6 to 10 days, 11 to 15 days, and >15 days were 22.53%, 31.33%, 21.67%, and 24.47%, respectively. The 3 most severe symptoms were cough (28.37%), body aches (22.37%), and fever (13.79), with 55.88% participants having severe symptoms lasting <5 days. Critically, 73.3% of infected staff required sick leave (mean 3.2 days), causing severe workforce shortages. Despite mild disease severity (low hospitalization), few underwent lab tests (10.5%), or computer tomography scans (8.2%); 92.52% infected patients received medications to alleviate symptoms, and the most common drugs included antipyretic and analgesic drugs (86.05%), antitussive drugs (56.97%), antiviral drugs (40.61%), and traditional Chinese medicine (33.01%). In summary, COVID-19 spread rapidly across hospitals in December 2022, resulting in high rate of absenteeism, demonstrating that even clinically mild outbreaks can critically disrupt healthcare capacity through staff depletion.

## 1. Introduction

Coronavirus disease (COVID-19), first identified in December 2019, rapidly evolved into a global pandemic.^[1,2]^ The causative agent, SARS-CoV-2, continuously mutated during the pandemic, generating variants of concern, which were classified as alpha (B.1.1.7), beta (B.1.351), gamma (P.1), delta (B.1.617.2), and omicron (B.1.1.529) by the World Health Organization.^[3]^ These mutations altered key viral characteristics, including transmissibility, disease severity, hospitalization risk, and mortality rates.^[4,5]^ While alpha, beta, gamma, delta, and omicron exhibited progressively enhanced transmissibility,^[6–8]^ alpha, beta, and delta variants were associated with increased risks of hospitalization and severe deaths.^[9,10]^ After November 2021, omicron became the dominant global variant. Compared to delta, omicron reduced hospitalization rates, lower intensive care unit admission, decreased need for mechanical ventilation, and shorter hospital stays.^[11–13]^

The symptoms vary across variants. Fever and cough remain the most common symptoms in adults.^[14]^ Although most patients infected with omicron variant are asymptomatic or mild, anosmia/ageusia have been observed in 1.3% of the patients.^[15]^ Notably, a significant proportion of adult symptoms remain uncharacterized in existing studies.^[14]^ While some studies involved medical personnel enabled precise symptom documentation,^[16,17]^ other studies also focused on mental health.^[18]^ Hospital staffs were asymptomatic when the COVID-19 epidemic ended. The diagnosis approaches of COVID-19 infection include nucleic acid detection, antigen test, as well as self-diagnosis (favored based on symptoms alone).^[19,20]^ Laboratory tests and computed tomography (CT) examinations have been used to assess the severity of the disease.^[21]^ When patients infected with COVID-19 do not require hospitalization, over-the-counter medications are often used to combat the virus and alleviate symptoms.^[22,23]^ However, it is unknown how hospital staff infected with COVID-19 are diagnosed and treated. Meanwhile, the pandemic’s early impact on healthcare workers was severe, its later-phase consequences remain unquantified.

Following China’s rapid surge in December 2022, this study aimed to investigate the symptoms patterns, diagnostic methods, treatment strategies, and operational impacts of COVID-19 on hospital staff during this period.

## 2. Methods

### 2.1. Subjects of the survey

All 1283 hospital staff at the Mianyang Central Hospital in Sichuan province were surveyed anonymously via Questionnaire Star APP, File S1, Supplemental Digital Content, https://links.lww.com/MD/P619, a professional online survey platform. As all responses were collected and analyzed anonymously, informed consent was waived. This study was approved by the Medical Ethics Committee of Mianyang Central Hospital (No. S20230327-02).

### 2.2. Research strategy

Based on a previous survey that investigated the knowledge of medical professionals, their practices, and their attitudes toward traditional Chinese medicine (TCM) for the prevention and treatment of COVID-19,^[24]^ we developed a self-reported questionnaire, File S1, Supplemental Digital Content, https://links.lww.com/MD/P619 for this study. The preliminary design of the questionnaire, File S1, Supplemental Digital Content, https://links.lww.com/MD/P619 was reviewed by 2 independent parties. The questions were evaluated to determine whether they successfully captured the topic, and a question formulation expert was consulted to verify whether the survey contained misleading, confusing, or double-barreled questions. We conducted a pilot test using a sample of 60 hospital workers, and based on the results of principal component analysis and internal consistency, we modified the questionnaire, File S1, Supplemental Digital Content, https://links.lww.com/MD/P619 accordingly. The reliability of the questionnaire, File S1, Supplemental Digital Content, https://links.lww.com/MD/P619 is (Cronbach alpha: 0.712). The questionnaire, File S1, Supplemental Digital Content, https://links.lww.com/MD/P619 was written in Chinese and consisted of 3 parts with a total of 15 questions. In the first part, participants were asked to provide their demographic data (e.g., sex and age). In the second part, participants were asked if they had been infected with COVID-19, and those infected with COVID-19 were further questioned on their symptoms and duration. In the third section, participants infected with COVID-19 were selected for the testing methods used for diagnosis and any drug treatments. All submitted responses were exported for analysis (100% response rate).

### 2.3. Data analysis

The results were analyzed using SPSS v22.0 (IBM Corp., Armonk, NY).^[25]^ Standard descriptive statistics were reported. The *χ*^2^-test was used to assess associations between demographic variables of those infected with COVID-19. The *χ*^2^-test was also is used for a univariate analysis of basic information and clinical treatment, and factors with a *P*-value < .05 were included in the multivariate multiple logistic regression analysis.^[26]^ A two-sided analysis was used, and a *P*-value < .05 was considered to indicate statistical significance.

## 3. Results

### 3.1. General characteristics of the participants and diagnostic methods

Table 1 presents the characteristics of the participants. Among 1464 hospital staff surveyed (452 male and 1012 female), 1283 participants were diagnosed with COVID-19. Diagnostic methods included: nucleic acid detection (14.5%), antigen testing (40.5%), and symptom-based self-diagnosis (45.1%). Table 2 shows the stratification of diagnostic methods by sex, age, and career status. A higher proportion of male participants (53.56%) chose self-diagnose based on symptoms. Participants under 41 years old (40.8%) were less likely to choose self-diagnosis based on symptoms compared to those over 41 years (52.6%), with the lowest rate observed in the 31 to 40 year age group (36.7%). Conversely, diagnosis via nucleic acid detection or antigen testing was more common among women (58.5%), participants under 41 (59.2%), and particularly those aged 31 to 40 (63.3%). Nurses (59.7%) were also more frequently diagnosed using nucleic acid detection or antigen testing.

**Table 1:** The characteristics of the participants.

	Infected 1283 (100%)	No infected 181 (100%)	*P*
Gender			<.001
Male	379 (29.54%)	73 (40.33%)	
Female	904 (70.46%)	108 (59.67%)	
Age (years)			<.001
21–30	402 (31.33%)	20 (11.05%)	
31–40	455 (35.47%)	29 (16.03%)	
41–50	207 (16.13%)	30 (16.67%)	
>50	219 (17.07%)	102 (56.35%)	
Career			<.001
Doctor	309 (24.08%)	5 (2.76%)	
Nurse	511 (39.84%)	18 (9.95%)	
Medical technology	55 (4.28%)	4 (2.21%)	
Administration	96 (7.48%)	7 (3.87%)	
Rear-service	312 (24.32%)	147 (81.21%)	

**Table 2:** Selection of diagnostic methods.

	Nucleic acid detection 182 (100%)	Antigen test 519 (100%)	Self-diagnosis based on symptoms 578 (100%)	*P*
Gender				<.001
Male	62 (33.33%)	114 (21.97%)	203 (35.12%)	
Female	124 (66.67%)	405 (78.03%)	375 (64.88%)	
Age (years)				<.001
21–30	67 (36.02%)	152 (29.29%)	183 (31.66%)	
31–40	61 (32.80%)	227 (43.73%)	167 (28.90%)	
41–50	25 (13.44%)	78 (15.03%)	104 (17.99%)	
>50	33 (17.74%)	62 (11.95%)	124 (21.45%)	
Career				<.001
Doctor	38 (20.43%)	125 (20.08%)	146 (25.26%)	
Nurse	70 (37.63%)	235 (45.28%)	206 (35.64%)	
Medical technology	15 (8.06%)	20 (3.86%)	20 (3.46%)	
Administration	14 (7.54%)	46 (8.86%)	36 (6.23%)	
Rear-service	49 (26.34%)	93 (17.92%)	170 (29.41%)	

### 3.2. The start time of symptoms of COVID-19

The symptoms of 52, 252, 667, 249, 48, and 5 participants start at December 1 to 5, 6 to 10, 11 to 15, 16 to 20, 21 to 25, and 26 to 30, most of the participants (91%) have been infected with COVID-19 within 15 days.

### 3.3. Symptoms of hospital staff infected with COVID-19 and duration of symptoms

Table 3 summarizes all symptoms and their duration, the most serious symptoms and their duration, and the severity of symptoms of hospital workers with COVID-19. The most common symptoms were cough (88.54%), fever (79.42%), body aches (73.03%), and expectoration (70.30%). The proportions of participants experiencing symptoms lasting <5 days, 6 to 10 days, 11 to 15 days, and >15 days were 22.53%, 31.33%, 21.67%, and 24.47%, respectively. The 3 most frequently reported severe symptoms were cough (28.37%), body aches (22.37%), and fever (13.79%). Overall, 55.88% participants had severe symptoms lasting <5 days. The proportions of mild, moderate and severe symptoms of hospital staff infected with COVID-19 were 42.25%, 57.05%, and 0.70%. Table S1, Supplemental Digital Content, https://links.lww.com/MD/P618 shows the number of days off-duty: 73.26% of hospital staff with COVID-19 were unable to work due to severe symptoms, with an average time off-duty of 3.2 days. Table 4 shows the univariate and multivariate analyses of the days off-duty in relation to the duration and severity of the most severe symptoms following COVID-19 diagnosis.

**Table 3:** The symptoms of hospital staff infected with COVID-19.

	Symptoms 1283 (100%)	Most serious symptoms 1283 (100%)
Fever	1019 (79.42%)	177 (13.79%)
Cough	1136 (88.54%)	364 (28.37%)
Expectoration	902 (70.30%)	70 (5.46%)
Pharyngalgia	857 (66.79%)	177 (13.80%)
Nasal obstruction	760 (59.23%)	46 (3.59%)
Runny nose	650 (50.66%)	15 (1.17%)
Olfactory disturbance	469 (36.55%)	11 (0.86%)
Gustatory disturbance	580 (45.22%)	16 (1.25%)
Diarrhea	268 (20.88%)	6 (0.47%)
Sick	246 (19.17%)	7 (0.55%)
Vomit	187 (14.57%)	6 (0.47%)
Dizzy	553 (43.10%)	25 (0.97%)
Headache	654 (50.97%)	65 (5.06%)
Chest distress	345 (26.89%)	22 (1.71%)
Cardio palmus	246 (19.17%)	21 (1.64%)
Body aches	937 (73.03%)	287 (22.37%)
Hearing loss	117 (9.12%)	6 (0.47%)
Others	73 (5.69%)	22 (1.71%)
Duration of time		
1–5 d	289 (22.53%)	717 (55.88%)
6–10 d	402 (31.33%)	225 (17.54%)
11–15 d	278 (21.67%)	125 (9.74%)
>15 d	314 (24.47%)	216 (16.84%)

**Table 4:** The univariate and multivariate analysis of time of days of out duty of hospital staff infected with COVID-19.

	Univariate analysis		Multivariate analysis	
Variables	*F*	*P*	*F*	*P*
Sex	24.285	<.001	0.132	0.717
Age	1.758	.153		
Career	4.154	.002	1.090	.360
Duration of symptoms	4.758	.003	0.516	.671
Duration of the most severe symptoms	6.472	<.001	5.158	.002
Severity of symptoms	145.787	<.001	32.028	<.001

### 3.4. Selection of laboratory and CT examinations

Only 10.52% and 8.18% of participants underwent laboratory testing and CT examinations, respectively. Table 5 shows the univariate and multivariate analyses for laboratory testing; the univariate analyses revealed that more male, doctors, nurses, participants with symptoms lasting ≥16 days, and those with severe symptoms chose laboratory tests. In the multivariate analyses, symptoms lasting ≥16 days and severe symptoms emerged as independent factors for selecting laboratory testing. Tables 6 shows the univariate and multivariate analyses of CT examinations; univariate analyses revealed that more participants aged 31 to 40 years, doctors, nurses, those with symptoms or most severe symptoms lasting ≥16 days, and participants with severe symptoms chose CT examinations. Multivariate analyses identified occupation, symptoms or most severe symptoms lasting ≥16 days, and severe symptoms as independent factors associated with the selection of CT examinations.

**Table 5:** The univariate and multivariate analysis of selection of laboratory testing.

	Univariate analysis		Multivariate analysis	
Variables	*χ* ^2^	*P*	*χ* ^2^	*P*
Sex	5.613	.018	2.294	.130
Age	0.313	.958		
Career	12.808	.012	7.582	.108
Duration of symptoms	18.663	<.001	14.050	.003
Duration of the most severe symptoms	6.259	.100		
Severity of symptoms	16.606	<.001	10.192	.006

**Table 6:** The univariate and multivariate analysis of selection of CT examinations.

	Univariate analysis		Multivariate analysis o	
Variables	*χ* ^2^	*P*	*χ* ^2^	*P*
Sex	0.454	.501		
Age	11.498	.009	0.708	.871
Career	31.522	<.001	21.507	<.001
Duration of symptoms	26.320	<.001	9.811	.020
Duration of the most severe symptoms	20.759	<.001	12.129	.007
Severity of symptoms	24.198	<.001	17.046	<.001

### 3.5. Treatment of hospital personnel infected with COVID-19

Table S2, Supplemental Digital Content, https://links.lww.com/MD/P618 lists the drugs used to treat hospital workers with COVID-19. The most common drugs included antipyretic and analgesic drugs (86.05%), antitussive drugs (56.98%), antiviral drugs (40.51%), and TCM (33.05%). Table 7 shows the univariate and multivariate analyses for antiviral drugs selection. The univariate analyses revealed that more nurses, administration staff and participants experiencing the most severe symptoms lasting ≥16 days chose antiviral drugs. Multivariate analyses identified the most severe symptoms lasting ≥16 days as an independent factor associated with antiviral drugs selection. Table 8 presents the univariate and multivariate analyses for antibacterial drugs selection. The univariate analyses revealed that more females, participants with symptoms or the most severe symptoms lasting ≥16 days, and participants with severe symptoms chose antibacterial drugs. Multivariate analyses identified the most severe symptoms lasting ≥16 days and severe symptoms as independent factors for antibacterial drugs selection. Table 9 outlines the analyses regarding TCM selection. The univariate found that more females and participants with symptoms or the most severe symptoms lasting ≥16 days chose TCM, and multivariate analyses identified the most severe symptoms lasting ≥16 days as an independent factor for TCM selection.

**Table 7:** The univariate and multivariate analysis of selection of antiviral drugs.

	Univariate analysis		multivariate analysis o	
Variables	*χ* ^2^	*P*	*χ* ^2^	*P*
Sex	4.977	.260		
Age	10.743	.013	2.864	.413
Career	64.448	<.001	58.255	<.001
Duration of symptoms	4.682	.197		
Duration of the most severe symptoms	8.251	.041	6.274	.099
Severity of symptoms	1.593	.451		

**Table 8:** The univariate and multivariate analysis of selection of antibacterial drugs.

	Univariate analysis		Multivariate analysis o	
Variables	*χ* ^2^	*P*	*χ* ^2^	*P*
Sex	6.350	.012	1.224	.747
Age	3.879	.275		
Career	9.120	.058		
Duration of symptoms	46.052	<.001	20.856	<.001
Duration of the most severe symptoms	33.068	<.001	16.095	.001
Severity of symptoms	10.359	.006	13.899	.001

**Table 9:** The univariate and multivariate analysis of selection of traditional Chinese medicine.

	Univariate analysis		Multivariate analysis o	
Variables	*χ* ^2^	*P*	*χ* ^2^	*P*
Sex	7.643	.006	1.839	.607
Age	1.439	.696		
Career	5.274	.260		
Duration of symptoms	34.409	<.001	18.802	<.001
Duration of the most severe symptoms	24.213	<.001	8.192	.042
Severity of symptoms	0.514	.773		

## 4. Discussion

This survey study provided insights into the symptoms, diagnosis, treatment, and impact on hospital personnel of COVID-19. The most common symptoms were cough, fever, body aches, and expectoration, all of which were similar to flu symptoms. Nonetheless, 9% of participants experienced hearing loss, and for 46.14% of the participants, symptom duration was over 10 days, which appears longer than typically reported for influenza. Moreover, almost none of the workers experienced fatigue or dyspnea, symptoms that were more prevalent in the early stages of the COVID-19 epidemic.^[14]^ The 3 most severe symptoms were cough, body aches, and fever; however, more than half of the participants had severe symptoms lasting <5 days. Approximately 73.27% participants were unable to work because of the duration of the most severe symptoms and the severity of the symptoms. Notably, only 0.7% of the participants required hospitalization for severe symptoms. This hospitalization rate appears substantially lower than rates reported in earlier phases of the pandemic.^[27–30]^ These results indicate that the virulence of COVID-19 is decreasing.

Laboratory tests and CT examinations have been used to diagnose and estimate the severity and prognosis of COVID-19.^[21,31,32]^ Participants with a longer duration of symptoms and more severe symptoms tended to undergo laboratory tests and CT examinations. Logistics staff with COVID-19 had the lowest CT examination rate, which may be related to the higher proportion of logistics staff reporting mild symptoms (42.2%) in this cohort. Diagnostic approaches differed by age and gender. Self-diagnosis based on symptoms was more common among male participants and those older than 41 years. In contrast, nucleic acid detection or antigen testing was more frequently used for diagnosis in women under 41 years. This pattern likely reflects the demographic composition of nursing staff (who predominantly used these tests and of whom 84.7% were under 41 years old).

Most of the participants were diagnosed with SARS-CoV-2 within 15 days, which was consistent with the characteristics of the omicron variant.^[6–8]^ Among all participants, the infection rate in women (89.3%) was higher than in men (83.8%), and the rate of COVID-19 in participants under 50 years of age (93.1%) was higher than that for participants over 50 years of age (68.2%). Logistics workers exhibited a lower infection rate compared to doctors (67.9% vs 98.4%) and nurses (67.9% vs 96.6%). This lower rate may be associated with potentially reduced exposure opportunities to infected patients. Within the logistics worker subgroup, higher infection rates were observed among males (43.6%) and individuals over 50 years of age (53.59%). Zhou et al reported that the positivity rate of hospital workers was obviously higher than that of healthcare workers and administrative staff in 2020, which may be due to differences between the early and late epidemic stages of COVID-19.^[33]^ In this study, more than 70% of the hospital staff with COVID-19 were unable to work; the average absence time was 3.2 days, which was shorter than that reported in other studies^[34,35]^ and likely contributed to a marked shortage of human resources. Participants with a longer duration of severe symptoms and those with more severe symptoms had a longer duration of absence from duty.

Most of the therapeutic agents that participants used to treat COVID-19 symptoms were antipyretics, analgesics, and antitussives. Only 16.1% participants with COVID-19 received antibacterial agents, whereas 57% and 33% received antiviral agents and TCM. The professions of the participants were related to the selection of antiviral drugs; fewer doctors (22%) and medical technical workers (32.7%) selected antiviral drugs than nurses (47.7%), administration (41.7%), or logistics workers (48.4%). Interestingly, among these professions, physicians were less likely to choose antiviral therapy, despite being the most knowledgeable about drugs. Participants with longer duration of symptoms or with more severe symptoms received antibacterial drugs. Instead, more participants with a longer duration of symptoms or with the most severe symptoms selected TCM for treatment. This is consistent with the findings of our previous survey, in which 62.1% of medical professionals agreed that TCM could alleviate symptoms of patients with COVID-19.^[24]^

While the world has now entered the post-pandemic period, epidemic diseases occur often. This study may provide some reference and alleviate emotions of panic during the next similar epidemic and provide assistance for hospitals regarding their response to future similar epidemics, including the organization of human resources.

## 5. Conclusions

In December 2022, COVID-19 spread rapidly across hospitals and nearly half of the staff self-diagnosed based on symptoms alone. Consequently, only a small number of participants underwent laboratory tests and CT examinations. Most of the symptoms of the participants were mild or moderate, resulting in very few hospitalizations. Most of the participants received treatment, primarily using symptomatic drugs, antivirals and TCM. Critically, despite the low pathogenicity of the coronavirus, COVID-19 still contributed to a shortage of human resources in hospitals.

## 6. Limitations and prospects

The present study had some limitations. First, our analysis was conducted in January 2023, while the data collection referred to December 2022; because of the forgetfulness of the participants, our data may deviate from the real-life situation. Second, 45.05% of the participants with COVID-19 were self-diagnosed, which may have led to an inaccurate number of diagnoses. Third, this was a single-center survey with a relatively small number of respondents.

## Author contributions

**Conceptualization:** Jing Pu, Xiaobo Du.

**Data curation:** Jing Pu, Mi Fan, Binwei Lin.

**Investigation:** Jing Pu, Mi Fan, Binwei Lin, Xiaobo Du.

**Methodology:** Jing Pu, Mi Fan, Binwei Lin.

**Project administration:** Xiaobo Du.

**Supervision:** Xiaobo Du.

**Writing – original draft:** Jing Pu.

**Writing – review & editing:** Xiaobo Du.

## Supplementary Material


