# Supplementary material for: Impact of COVID-19 hospitalization on December 2022 hospital staff in Sichuan, China
Source: Medicine (Baltimore). 2025 Aug 8;104(32):e43784. doi: 10.1097/MD.0000000000043784 (PMC12338257; doi:10.1097/MD.0000000000043784)
Supplement: Supplementary file 1 [file medi-104-e43784-s001.docx]

**Survey Questionnaire on Symptoms Following COVID-19 Infection in Healthcare Workers**

Hello! Thank you very much for participating in our survey. This questionnaire aims to assess the symptoms experienced by our healthcare workers following COVID-19 infection. We adhere to relevant ethical principles to protect personal information from disclosure. Data will be used solely for scientific research purposes. There are no right or wrong answers to the questions; please answer honestly based on your personal experience. Thank you!

**Part 1: Basic Information**

1.1 Your Gender: [Single Choice] *
( ) Male
( ) Female

1.2 Your Age Group: [Single Choice] *
( ) 21-30 years
( ) 31-40 years
( ) 41-50 years
( ) 51 years and above

1.3 Your Occupation: [Single Choice] *
( ) Doctor
( ) Nurse
( ) Medical technology
( ) Administration

( ) Rear-service

**Part 2: Symptoms**

2.1 Have you been infected with COVID-19 within the past month? [Single Choice] *
*(If "Yes", please complete the remainder of Part 2 and Part 3)*
( ) Yes (Diagnosed by Nucleic acid detection )
( ) Yes (Diagnosed by Antigen test)
( ) Yes (Self-diagnosed based on symptoms)
( ) No

2.2 If infected, which symptoms did you experience? [Multiple Choice] *
□ Fever
□ Cough

□ Expectoration
□ Pharyngalgia
□ Nasal obstruction

□Runny nose
□ Olfactory disturbance

□ Gustatory disturbance
□ Diarrhea
□ Vomit
□ Dizzy

□ Headache

□ Chest distress

□ Cardio palmus

□ Body aches
□ Hearing loss
□ Other symptoms: _______________

2.3 Total duration of ALL symptoms experienced:
( ) ≤ 5 days
( ) 6-10 days
( ) 11-15 days
( ) > 15 days

2.4 Your MOST SEVERE symptom was: [Single Choice] *
□ Fever
□ Cough

□ Expectoration
□ Pharyngalgia
□ Nasal obstruction

□Runny nose
□ Olfactory disturbance

□ Gustatory disturbance
□ Diarrhea
□ Vomit
□ Dizzy

□ Headache

□ Chest distress

□ Cardio palmus

□ Body aches
□ Hearing loss
□ Other symptoms: _______________

2.5 Duration of your MOST SEVERE symptom:
( ) ≤ 5 days
( ) 6-10 days
( ) 11-15 days
( ) > 15 days

2.6 Severity of your symptoms: [Single Choice] *
( ) Mild (Able to work)
( ) Moderate (Unable to work, required rest)
( ) Severe (Required hospitalization)

2.7 Number of workdays missed due to symptom severity: ________ days

**Part 3: Laboratory Investigations and Treatment**

3.1 During this infection episode, did you undergo any laboratory tests (e.g., body fluid or blood tests)? [Single Choice] *
( ) Yes
( ) No

3.2 During this infection episode, did you undergo a CT scan? [Single Choice] *
( ) Yes
( ) No

3.3 During this infection episode, did you receive pharmacological treatment? [Single Choice] *
*(If "Yes", please answer 3.4)*
( ) Yes
( ) No

3.4 During this infection episode, which medications did you use? [Multiple Choice] *
□ Antipyretics/Analgesics (e.g., Paracetamol, Ibuprofen)
□ Antiviral drugs (e.g., Paxlovid, Molnupiravir)
□ Antibacterial drugs (Antibiotics)
□ Traditional Chinese Medicine (TCM) / Chinese patent herbal medicines
□ Antitussives (Cough suppressants/expectorants)
□ Antidiarrheal drugs
□ Corticosteroids
□ Other: _______________
