# Supplementary material for: Impact of COVID-19 hospitalization on December 2022 hospital staff in Sichuan, China
Source: Medicine (Baltimore). 2025 Aug 8;104(32):e43784. doi: 10.1097/MD.0000000000043784 (PMC12338257; doi:10.1097/MD.0000000000043784)
Supplement: Supplementary file 2 [file medi-104-e43784-s002.docx]

Supplement Table 1 The days of out duty of hospital staff infected with COVID-19

| Days | Participants |
| --- | --- |
| 0 day | 343 |
| 0.5 day | 9 |
| 1 day | 117 |
| 1.5 days | 3 |
| 2 days | 202 |
| 2.5 days | 1 |
| 3 days | 256 |
| 3.5 days | 1 |
| 4 days | 96 |
| 5 days | 139 |
| 6 days | 24 |
| 7 days | 60 |
| 8 days | 4 |
| 10 days | 18 |
| 11 days | 2 |
| 14 days | 3 |
| 15 days | 3 |
| 30 days | 2 |

Supplement Table 2 The selection of drugs of hospital staff infected with COVID-19.

|  | Yes | No |
| --- | --- | --- |
| Drugs | 1187 | 96 |
| Antipyretic and analgesic | 1014 | 269 |
| Antiviral | 521 | 762 |
| Antibacterial | 207 | 1076 |
| Traditional Chinese medicine | 424 | 859 |
| Antitussive | 731 | 552 |
| Antidiarrheal | 43 | 1240 |
| Corticosteroids | 68 | 1215 |
| Others | 35 | 1248 |
